# Reactivation of Cutaneous Leishmaniasis after Renal Transplantation: A Case Report

**DOI:** 10.1155/2014/251423

**Published:** 2014-01-16

**Authors:** Hossein Mortazavi, Mehrnaz Salehi, Kambiz Kamyab

**Affiliations:** ^1^Department of Dermatology, Tehran University of Medical Sciences, Razi Hospital, Vahdat Islamic Square, Tehran 1199663911, Iran; ^2^Department of Pathology, Tehran University of Medical Sciences, Razi Hospital, Vahdat Islamic Square, Tehran 1199663911, Iran

## Abstract

A 45-year-old man with reactivation of previously existing and subsiding cutaneous leishmaniasis on his wrist and lower leg (shin) after renal transplantation was admitted to our dermatology service on March 2008. He presented to us with two huge tumoral and cauliflower-like lesions. Skin smear and histopathology of skin showed leishman bodies and confirmed the diagnosis. After renal transplantation, he received cyclosporine plus prednisolone to induce immunosuppression and reduce the probability of transplant rejection. After immunosuppressive therapy, reactivation of cutaneous leishmaniasis with the above presentation took place. The patient responded to 800 mg/day intravenous sodium stibogluconate for 3 weeks plus local cryotherapy. Systemic plus local therapy along with reducing the doses of immunosuppressive drugs led to improvement of lesions. Reactivation of leishmaniasis after immunosuppression has been rarely reported.

## 1. Introduction

Cutaneous leishmaniasis (CL) is caused by a parasite from the genus *Leishmania* infection and is transmitted to humans by (female) sand flies bite [1]. In general, reactivation of CL occurs due to immunosuppression. Environmental factor and aging may also lead to reactivation of CL [2].

Herein, we present a patient with reactivation of CL after renal transplantation and immunosuppressive therapy who responded to a combination of intravenous (IV) sodium stibogluconate plus local therapy and reducing doses of immunosuppressive drugs.

## 2. Case Report

A 45-year-old Iranian man with renal transplant and with two tumoral lesions was admitted to our dermatology ward on March 2008. The patient was suffering from chronic renal failure due to diabetes (non-insulin-dependent diabetes mellitus, NIDDM) for several years before this admission.

The patient developed small popular lesion (2 × 5 millimeter) on his left wrist and right leg (shin) after traveling to an endemic leishmaniasis area (Natanz in Isfahan province, Iran) in April 2006. The diagnosis of CL was confirmed by a positive direct smear for leishman bodies. As the lesions were small, they remained untreated to obtain immunity for the patient.

**Figure 1:**
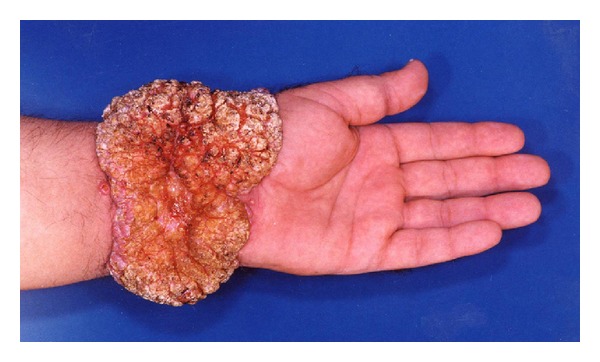
Tumoral and cauliflower-like lesion on the left wrist.

**Figure 2:**
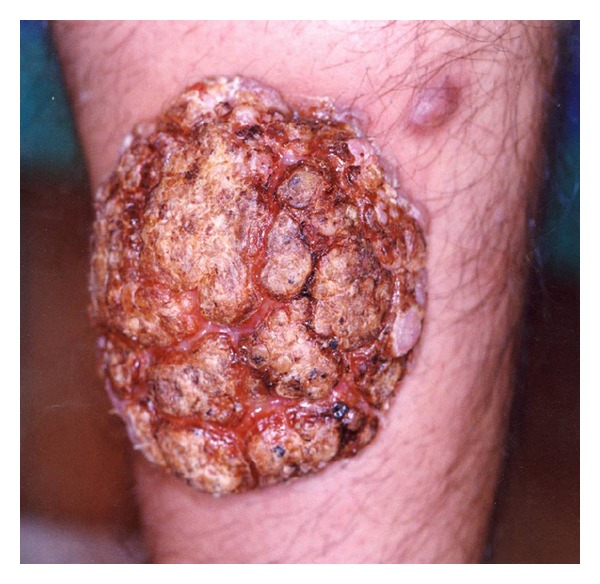
Tumoral lesion on the right shin.

Due to chronic renal failure, renal transplantation was performed on the patient in August 2007. After transplantation, he received prednisolone tablets 50 mg/day and cyclosporine 7.5 mg/kg daily (equivalent to 600 mg for an 80 kg patient). Several weeks after renal transplantation and immunosuppressive therapy, these small popular lesions became large cauliflower-like and tumoral lesions measured 3 × 4 × 5 cm on his left wrist and 4 × 5 × 6 cm on his right leg (shin) (Figures 1 and 2).

A slit-skin smear showed leishman bodies in Giemsa preparations. The leishmanin skin test (Montenegro) was positive with 5 mm induration.

**Figure 3:**
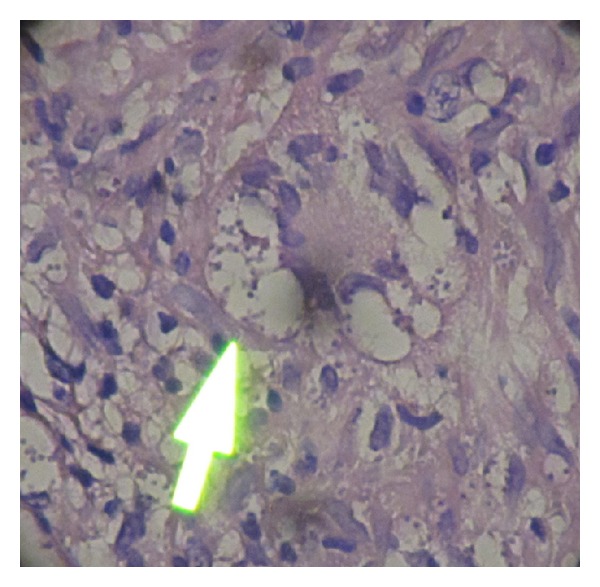
Leishman body in H&E staining slide of the lesion.

Histopathological examination of the biopsy obtained specimens demonstrated ulcerative changes and irregular acanthosis in the epidermis, namely, pseudoepitheliomatous hyperplasia. In dermis infiltration of lymphocyte, plasma cell and histiocyte with multinucleated Giant cell were shown. Leishman bodies were seen in histiocytes, especially in the specimen obtained from the lower leg lesion (Figure 3). With regard to the clinical and histopathological findings, the diagnosis of reactivation of CL was made.

After admission to our dermatology department, he was treated with 800 mg/day IV sodium stibogluconate (Pentostam, pentavalent antimonials) for 3 weeks. In addition, his lesions were treated with local destructive cryotherapy; cryotherapy was applied by using the liquid nitrogen according to Asilian et al. [3]. The patient responded favorably to the above treatment. Afterward, the patient was followed up for 5 years. At the latest visit on June 2013, the direct smear and polymerase chain reaction from the site of previous lesion were negative for leishman body.

## 3. Discussion

Clinical and histopathological features of our patient (with enlargement of lesions after renal transplantation and fairly too many numbers of leishman bodies in direct smear and hematoxylin and eosin (H&E) staining slides of the patient) were in favor of the diagnosis of reactivation of CL. The diagnosis of *Leishmania* recidiva cutis was ruled out, since this entity is usually characterized by small yellowish-brown nodules on the face which have an apple-jelly appearance on diascopy. In *Leishmania* recidiva cutis, worsening of lesions usually occurs in summer, especially in Iran [4, 5]. In addition, the histopathology of *Leishmania* recidiva cutis shows granulomatous reaction pattern with sparse plasma cell. Leishman body is also sparse or absent in a microscopic field [6].

In a report from England, dated 1999, an 85-year-old man who developed CL on his face after cutaneous surgery was presented [7]. He was a native English man who traveled to endemic areas, namely, Iran, Iraq, Lebanon, and Egypt, 50 years before the presentation of his lesions. Indeed, he had a reactivation of a dormant parasitic infection after 50 years [7].

In 2006, Mirzabeigi et al. reported reactivation of cutaneous leishmaniasis in a female patient after kidney transplantation [8]. The patient had a dormant *Leishmania* infection prior to renal transplantation. After surgery, she was on immunosuppressive drugs including tacrolimus, methyl prednisolone, and epoetin-*α*. Two months after transplantation, multiple nonulcerated nodules developed on both of her lower extremities. The H&E stain prepared pathology slide revealed a lymphohistiocytic inflammation. In the Giemsa stained slide, amastigotes were shown in histiocytes. In this patient, reactivation of cutaneous leishmaniasis was due to high doses of immunosuppressive drugs after transplantation [8].

Recently, a case of dissemination of localized cutaneous leishmaniasis after renal transplantation was reported from Fars province, an endemic region of leishmaniasis in Iran. This patient had diabetes type 2 and after surgery received an immunosuppressive regimen including mycophenolate mofetil, tacrolimus, and prednisolone to prevent rejection of the transplant [9]. Reactivation of American cutaneous leishmaniasis after treatment with corticosteroids for coincident rheumatologic disease has been reported as well [10].

An unusual clinical presentation of CL presenting as mucosal leishmaniasis has also been reported in a renal transplant patient with long-term immunosuppressive therapy from Italy [11].

In 1979, a case of fatal visceral leishmaniasis after renal transplantation was reported. This patient had been treated with prednisone and azathioprine for prevention of graft rejection [12]. In another study, reactivation of two cases of visceral leishmaniasis was reported [13].

In an animal study in BALB/c mice chronically infected with *Leishmania infantum*, reactivation of visceral leishmaniasis after long-term administration of glucocorticosteroid was observed. In this study, the immunosuppression induced by dexamethasone caused reactivation of *Leishmania* proliferation in the spleen of the animal [14].

In another animal study, Mendez et al. have shown that T regulatory cells (CD4–CD25) are involved in reactivation of leishmaniasis. They also showed the increased number of T regulatory cells at the site of reactivation [2].

In summary, reactivation of visceral and cutaneous leishmaniasis has been reported in several studies [7–9, 12].

In the present study, immunosuppression due to immunosuppressive drugs (cyclosporine plus prednisolone) is considered to be the main cause of reactivation of CL.

It has been shown that nitric oxide (NO) has a major role in the cell-mediated immune response of host against intracellular parasites [11, 15]. Both prednisolone and cyclosporine have effect on reduction of Th1 cell response, which is required for the improvement of CL. On the other hand, suppression of NO and reduction in IL-2 and IFN-*γ* due to immunosuppressive therapy would contribute to the susceptibility of patients to infections and its reactivation [11].

Treatment of CL with sodium stibogluconate plus local destructive therapy along with reducing the doses of immunosuppressive drugs led to improvement of lesions. After treatment, the patient was followed up for 5 years with no recurrence of lesions. Sodium stibogluconate: Pentostam.

## References

[1] Ponte-Sucre A, Ponte-Sucre A, Diaz E, Padrón-Nieves M (2013). Introduction: leishmaniasis—the biology of a parasite. *Drug Resistance in Leishmania Parasites*.

[2] Mendez S, Reckling SK, Piccirillo CA, Sacks D, Belkaid Y (2004). Role for CD4^+^ CD25^+^ regulatory T cells in reactivation of persistent Leishmaniasis and control of concomitant immunity. *Journal of Experimental Medicine*.

[3] Asilian A, Sadeghinia A, Faghihi G, Momeni A (2004). Comparative study of the efficacy of combined cryotherapy and intralesional meglumine antimoniate (Glucantime) versus cryotherapy and intralesional meglumine antimoniate (Glucantime) alone for the treatment of cutaneous leishmaniasis. *International Journal of Dermatology*.

[4] Cannavò SP, Vaccaro M, Guarneri F (2000). Leishmaniasis recidiva cutis. *International Journal of Dermatology*.

[5] Pettit JHS, Parish LC (1984). *Manual of Tropical Dermatology*.

[6] Weedon D (2010). *Weedon's Skin Pathology*.

[7] Czechowicz RT, Millard TP, Smith HR, Ashton RE, Lucas SB, Hay RJ (1999). Reactivation of cutaneous leishmaniasis after surgery. *British Journal of Dermatology*.

[8] Mirzabeigi M, Farooq U, Baraniak S, Dowdy L, Ciancio G, Vincek V (2006). Reactivation of dormant cutaneous Leishmania infection in a kidney transplant patient. *Journal of Cutaneous Pathology*.

[9] Zandieh A, Zandieh B, Dastgheib L (2013). Dissemination of localized cutaneous leishmaniasis in an organ transplant recipient: case report and literature review. *International Journal of Dermatology*.

[10] Tuon FF, Amato VS, Floeter-Winter LM (2007). Cutaneous leishmaniasis reactivation 2 years after treatment caused by systemic corticosteroids - First report. *International Journal of Dermatology*.

[11] Borgia F, Vaccaro M, Guarneri F, Manfrè C, Cannavò SP, Guarneri C (2001). Mucosal leishmaniasis occurring in a renal transplant recipient. *Dermatology*.

[12] Broeckaert-van Orshoven A, Michielsen P, Vandepitte J (1979). Fatal leishmaniasis in renal-transplant patient. *The Lancet*.

[13] Simon I, Wissing KM, Del Marmol V (2011). Recurrent leishmaniasis in kidney transplant recipients: report of 2 cases and systematic review of the literature. *Transplant Infectious Disease*.

[14] Rousseau D, Suffia I, Ferrua B, Philip P, Le Fichoux Y, Kubar J (1998). Prolonged administration of dexamethasone induces limited reactivation of visceral leishmaniosis in chronically infected BALB/c mice. *European Cytokine Network*.

[15] Guarneri C, Vaccaro M, Cannavò SP, Borgia F, Guarneri B (2002). Erythematous-edematous-infiltrative plaque on the face: cutaneous angio-lupoid leishmaniasis. *European Journal of Dermatology*.

